# Embracing the Evolution of Pharmacy Practice by Empowering Pharmacy Technicians

**DOI:** 10.3390/pharmacy8020066

**Published:** 2020-04-15

**Authors:** Ryan Burke

**Affiliations:** Pharmacy Technician Certification Board, Washington, DC 20037, USA; rburke@ptcb.org

**Keywords:** pharmacy technicians, pharmacy workforce, certification, education, regulations, board of pharmacy

## Abstract

While pharmacy technician roles in some practice settings are expanding beyond the traditional dispensing activities to include advanced or specialized tasks such as immunization administration, medication history collection, and final product verification, these practices are not yet widespread. There are apparent barriers to expanding the role of pharmacy technicians, including inconsistency in the education, training, and certification requirements across the United States, and regulations that have not kept pace with the evolving role of pharmacy technicians. Every corner of the profession has an opportunity, and responsibility, to elevate pharmacy technicians in an effort to advance safety and better serve patients. Regulators can expand the responsibilities that may be delegated to technicians, professional organizations can bring pharmacy technicians into the fold, employers can build career ladders to allow for advancement, and individual pharmacists and pharmacy technicians can advocate and engage.

Pharmacy technicians are the backbone of pharmacies across the globe. In the United States (U.S.), there are more than 420,000 pharmacy technicians in the workforce and the occupation is expected to grow by 7% during the next 10 years. Pharmacy technicians work under the supervision of pharmacists and are typically responsible for tasks such as data entry, medication dispensing, inventory management, insurance claims processing, and customer service [1]. While technicians’ roles in some practice settings are expanding beyond the traditional activities to include advanced or specialized tasks such as immunization administration, medication history collection, and final product verification, these practices are not yet widespread. The pharmacy profession has an opportunity to embrace the evolution of practice and determine how to best position pharmacy technicians to meet the public’s health care needs and enable pharmacists to fully utilize their clinical knowledge and skills. Achieving this in a comprehensive way requires all stakeholders, from regulators and professional associations to individual pharmacy technicians and pharmacists, to engage and take action.

Research supports the safety and efficacy of advanced pharmacy technician roles. Studies have demonstrated that the implementation of technician product verification in institutional and community pharmacy settings is a safe and effective way to allow pharmacists to focus on direct patient care. Frost and Adams summarized the findings of four community, pharmacy-focused studies that revealed trained pharmacy technicians perform product verification as accurately as pharmacists [2]. In another study by Hohmeier and colleagues, implementation of a practice model in which pharmacy technicians were responsible for product verification resulted in pharmacists spending significantly more time delivering patient care services [3].

Similarly, studies focused on medication history collection have found that pharmacy technicians are accurate and time efficient and, in some cases, may also decrease costs [4,5]. In terms of immunization administration, McKeirnan and Sarchet have affirmed that appropriately trained pharmacy technicians improve access to vaccination care, thereby having the potential to increase the number of immunizations given and reduce the number of deaths from vaccine-preventable diseases [6]. 

There are two apparent barriers to expanding the role of pharmacy technicians: inconsistency in the education, training, and certification requirements across the U.S., and regulations that have not kept pace with the evolving roles of pharmacy technicians. 

Notably, there is stark disparity between consumers’ high expectations for technician training and the reality of pharmacy practice, suggesting public support for more consistent requirements for technician education and credentialing to advance safety. A 2016 public perception survey revealed that U.S. consumers view pharmacy technician education, training, and certification requirements as critically important. In fact, 94% said their trust in pharmacy would increase with standardized certification for pharmacy technicians; 88% say it is very important for people who compound or mix custom medications to be specially trained and certified; 77% of respondents said states should require all pharmacy technicians to be trained and certified, and 76% said they would seek out a different pharmacy if they knew technicians working in their current pharmacy were not certified [7].

The reality is that state regulations for education, training, and certification vary widely, thus creating a barrier to advancing technician roles. There are 45 states that require pharmacy technicians to be registered or licensed by the state’s board of pharmacy, and while 22 states require technicians to obtain national certification, only nine of those states require them to maintain certification throughout their careers.

There are inherent benefits to national pharmacy technician certification. Wheeler and colleagues recently compared the viewpoints of certified and noncertified technicians and explored the perceived value of certification in the areas of medication safety, skills and abilities, experience, career engagement and satisfaction, and productivity. They found that certified technicians have a stronger commitment to their careers and employers, a perceived lower rate of medication errors, and a stronger desire to take on new roles than technicians who are not certified [8]. In recent focus groups, Desselle and colleagues concluded that national certification has a positive impact on technician maturation, professional socialization, and career commitment [9]. 

In some states, national certification enables pharmacy technicians to perform certain tasks that noncertified technicians are prohibited from performing. For example, in Ohio, certified pharmacy technicians may accept verbal prescription orders, transfer prescriptions, and perform sterile compounding while those without certification may not [10].

In terms of pharmacy technician education and training, there are two accreditation standards: one created jointly by the American Society of Health-System Pharmacists and the Accreditation Council for Pharmacy Education (ASHP/ACPE) [11], and the other created by the Accrediting Bureau for Health Education Schools (ABHES) [12]. Aside from these accrediting organizations, both national pharmacy technician certification organizations require completion of an education and training program or work experience as part of their certification program’s eligibility criteria [13,14]. To date, only two states, North Dakota and Louisiana, have adopted accredited education and training as the entry-level requirement for all pharmacy technicians in the state. Illinois and Virginia are pursuing legislative and regulatory changes to implement similar requirements in their respective states. 

The second primary barrier to expanding technician roles is that, despite the evidence, many states’ current regulations do not enable pharmacy technicians to take on advanced or specialized tasks. This must change if the profession is to fully realize the benefits of a trained, certified, and committed pharmacy technician workforce. There has been some progress on this front. Two states, California and New Hampshire, are aiming to create an advanced pharmacy technician license category that would have different requirements and privileges than a standard technician registration or license [15,16]. In other places, like Idaho, the board of pharmacy has taken a less prescriptive approach by allowing pharmacists to use their professional judgment to determine which tasks to delegate to pharmacy technicians [17]. 

Today, 18 states allow technician product verification in some form, though in some cases it is limited to specific processes in the institutional setting (e.g., filling or replenishing an automated dispensing cabinet). While only three states currently allow technicians to administer immunizations, several states are actively exploring this task, and it is reasonable to expect more states to follow suit in the coming years.

While research and regulatory changes are important, pharmacy technicians also need to be engaged and prepare for new roles. This may come in the form of professional development through their employers or by seeking advanced or specialized training and credentials. Likewise, both pharmacy technicians and pharmacists have a responsibility to advocate for change through state or national professional associations and by voicing their opinion at state board of pharmacy meetings. 

Twelve states have taken the positive step of appointing pharmacy technicians to serve on the state board of pharmacy. Other states should consider this approach to ensure pharmacy technicians have a voice in the critical regulatory process. 

While some professional associations, such as ASHP, have a robust pharmacy technician membership focused on issues of importance to those professionals, many do not [18]. Anecdotally, some pharmacy technicians question the value in organizations that seek to represent them but have a name that implies exclusivity (i.e., “pharmacists”). Professional organizations should consider how their governance structures, membership models, messaging, and policy positions can be changed to better include and represent pharmacy technician viewpoints. 

The pharmacy profession is evolving at a rapid pace. Every corner of the profession has an opportunity, and responsibility, to elevate pharmacy technicians in an effort to advance safety and better serve patients. Regulators can expand the responsibilities that may be delegated to technicians, professional organizations can bring pharmacy technicians into the fold, employers can build careers ladders to allow for advancement, and individual pharmacists and pharmacy technicians should advocate and engage.
